# pH and microbial community determine the denitrifying activity in the presence of nitrate-containing radioactive waste

**DOI:** 10.3389/fmicb.2022.968220

**Published:** 2022-10-21

**Authors:** Kristel Mijnendonckx, Nele Bleyen, Axel Van Gompel, Ilse Coninx, Natalie Leys

**Affiliations:** ^1^Unit of Microbiology, SCK CEN, Mol, Belgium; ^2^W&D Expert Group, SCK CEN, Mol, Belgium; ^3^Biosphere Impact Studies, SCK CEN, Mol, Belgium

**Keywords:** nitrate reduction, alkaline pH, Harpur Hill sediment, Boom Clay borehole water, bitumen

## Abstract

An important fraction of the currently stored volume of long-lived intermediate-level radioactive waste in Belgium contains large amounts of NaNO_3_ homogeneously dispersed in a hard bituminous matrix. Geological disposal of this waste form in a water-saturated sedimentary formation such as Boom Clay will result in the leaching of high concentrations of NaNO_3_, which could cause a geochemical perturbation of the surrounding clay, possibly affecting some of the favorable characteristics of the host formation. In addition, hyper-alkaline conditions are expected for thousands of years, imposed by the cementitious materials used as backfill material. Microbial nitrate reduction is a well-known process and can result in the accumulation of nitrite or nitrogenous gases. This could lead to the oxidation of redox-active Boom Clay components, which could (locally) decrease the reducing capacity of the clay formation. Here, we compared nitrate reduction processes between two microbial communities at different pH related to a geological repository environment and in the presence of a nitrate-containing waste simulate during 1 year in batch experiments. We showed that the microbial community from in Boom Clay borehole water was able to carry out nitrate reduction in the presence of acetate at pH 10.5, although the maximum rate of 1.3 ± 0.2 mM NO_3_^−^/day was much lower compared to that observed at pH 9 (2.9 mM NO_3_^−^/day). However, microbial activity at pH 10.5 was likely limited by a phosphate shortage. This study further confirmed that the Harpur Hill sediment harbors a microbial community adapted to high pH conditions. It reduced twice as much nitrate at pH 10.5 compared to pH 9 and the maximum nitrate reduction rate was higher at pH 10.5 compared to that at pH 9, i.e., 3.4 ± 0.8 mM NO_3_^−^/day versus 2.2 ± 0.4 mM NO_3_^−^/day. Both communities were able to form biofilms on non-radioactive Eurobitum. However, for both microbial communities, pH 12.5 seems to be a limiting condition for microbial activity as no nitrate reduction nor biofilm was observed. Nevertheless, pH alone is not sufficient to eliminate microbial presence, but it can induce a significant shift in the microbial community composition and reduce its nitrate reducing activity. Furthermore, at the interface between the cementitious disposal gallery and the clay host rock, the pH will not be sufficiently high to inhibit microbial nitrate reduction.

## Introduction

Microbial nitrate reduction at near neutral or slightly alkaline pH is a well-known process (Libert et al., 1997; Bleyen et al., 2017, 2018a; Mijnendonckx et al., 2020). However, microbial nitrate reduction at highly alkaline conditions is less well studied (Albina et al., 2019). In general, alkaline conditions hamper microbial processes as the lack of protons likely impedes the proton motive force needed by most respiring microorganisms to synthesize adenosine triphosphate (ATP) and it interferes with the ability to maintain a circumneutral intracellular pH (Horikoshi and Akiba, 1982; Jin and Kirk, 2018). Moreover, pH can influence nutrient dissolution or precipitation and geochemical reactions, hence affecting the concentration of nutrients available for microbial growth (Jin and Kirk, 2018). One of the environments where microorganisms will be exposed to hyper alkaline conditions is during geological disposal of radioactive waste. Geological disposal implies isolating the waste from the surface by a combination of multiple engineered barriers and a stable geological formation (Alexander et al., 2015). In many geological disposal concepts, hyper alkaline conditions (pH > 12.5) are expected for thousands of years, imposed by the cementitious materials used as backfill material or as immobilization matrix (Phung et al., 2017).

Bitumen has been used as a matrix for the immobilization of precipitation or evaporator sludge, ion-exchange resins, liquid concentrates, incinerator ashes and filter materials since the late 1960’s (IAEA, 1993). Eurobitum, an important bituminized intermediate-level long-lived radioactive waste form in Belgium, contains soluble and insoluble salts with NaNO_3_ (20–30 wt%) and CaSO_4_ (4–6 wt%) being the most important (Valcke et al., 2000b). For this type of waste, the Belgian radioactive waste management organization, ONDRAF/NIRAS, currently envisages geological disposal in a poorly indurated clay as the reference long-term management option. Geological disposal of this waste in a water-saturated sedimentary formation such as the Belgian Boom Clay will induce water uptake by the hygroscopic salts present in the bituminized waste. This results in the dissolution and diffusion-controlled leaching of NaNO_3_ and other soluble salts. The resulting nitrate plume could cause a geochemical perturbation of the surrounding clay, possibly affecting the redox conditions, causing ionic strength effects and cation exchange processes, which might result in an increased mobility of the radionuclides through the host formation (Bleyen et al., 2018b; Valcke et al., 2022). Furthermore, the bitumen matrix will be continuously exposed to chemical and radiolytical degradation, resulting in the production of gases with hydrogen being the most important. Next to the production of gases, degradation triggers the leaching of water-soluble organic degradation products. Some of these may form complexes with radionuclides, thereby increasing their solubility and decreasing their sorption (Van Loon and Kopajtic, 1990; Valcke et al., 2000a; Walczak et al., 2001). The type and concentration of the degradation products is dependent on the (radio)chemical conditions. Nevertheless, some of the most prominent anoxic organic bitumen degradation products are acetate and formate, while oxalate is only observed as radiolytic degradation product in oxic conditions (Van Loon and Kopajtic, 1990; Kagawa et al., 2000; Libert and Walczak, 2000; Walczak et al., 2001; Valcke et al., 2022).

The impact of possible purely chemical reactions between nitrogen compounds and redox-active compounds of Boom Clay is expected to be limited (Bleyen et al., 2016b, 2018b; Hendrix et al., 2022). Contrary, leached nitrate could stimulate nitrate-reducing microorganisms. Microbial nitrate reduction could result in an accumulation of nitrite, which could lead to the oxidation of redox-active Boom Clay components and could thus (locally) decrease the reducing capacity of the clay formation (Parmentier et al., 2014; Bleyen et al., 2016b, 2018b). Complete denitrification to nitrogen gases could lead to the formation of a separate gas phase, if the concentration of produced N gases would exceed the solubility limit of the gases. As clay formations are very gas-tight, gas evacuation is slow and an excessive gas pressure increase might cause fissuring of the host rock, which would result in the formation of preferential pathways for radionuclide migration (Mallants et al., 2007; Harrington et al., 2012). On the other hand, hydrogen-dependent nitrate reduction results in a net gas consumption. Moreover, it has been shown that ammonium is formed during hydrogen-dependent nitrate reduction (Bleyen et al., 2017). Ammonium can sorb onto clay minerals and therefore compete with some radionuclides for sorption (e.g., Ni, Pd, Cs). It could also affect the solubility of certain radionuclides (e.g., Pd, Ni; Van Loon and Kopajtic, 1991; Staunton et al., 2002; Missana et al., 2004; Mihara et al., 2011).

Although extremely alkali-tolerant microorganisms have been identified in the past (Takai et al., 2001; Roadcap et al., 2006; Shapovalova et al., 2008), there is no consensus regarding the upper limit for microbial life. Studying microbial activity at alkaline pH in batch experiments while maintaining such high pH conditions throughout the entire experiment is an important experimental challenge (Sorokin, 2005). Recently, a 234-day long experiment investigated microbial nitrate reduction at initial pH 12.5. Microbial nitrate reduction with acetate as electron donor was only observed after manually lowering the pH to pH 11 at day 195 (Albina et al., 2021). The study added a single nitrate concentration and no solid fraction was present to enable biofilm formation, which could putatively enhance denitrification rates in alkaline environments (Alquier et al., 2014; Rafrafi et al., 2017). To further understand microbial processes at high pH several natural analogues sites are investigated. In the Maqarin natural analogue (with a maximum pH of 12.9), only slow growth and generally low metabolic activity of alkaliphiles were found (Pedersen et al., 2004). On the other hand, an active microbial population was found in an alkaline lake in the US at pH ~12.5 (Roadcap et al., 2006). One well-known natural analogue is the Harpur Hill site near Buxton, UK, that has been contaminated for decades by waste from a legacy lime works. The continuous percolation of residual lime (CaO) within the lime waste deposited at the site, by rainwater and shallow groundwater, has led to the development of an alkaline, calcium hydroxide dominated leachate with a pH of about 12.5 and the formation of an alkaline ‘lagoon’ of calcium carbonate tufa precipitate. However, the pH of the fluids at the site varies drastically, and is influenced by factors such as rainfall. A continued calcium carbonate deposition and pH reduction occurs through mixing with fresh water inflows and uptake of atmospheric CO_2_ as the surface water flows downstream. Sediments comprise high calcium and silicate concentrations and are seen as analogous to some conditions present in a cementitious geological disposal facility (Milodowski et al., 2013). A multitude of studies on Harpur hill sediments has been performed to further understand microbial processes in a cementitious waste repository and this resulted in the isolation of several new microbial species (Rizoulis et al., 2012; Bassil et al., 2015; Kuippers et al., 2015; Rout et al., 2017; Bassil and Lloyd, 2019; Charles et al., 2019; Byrd et al., 2021). Microbial nitrate and iron reducing activity occurred up to pH 11, while degradation of organic matter only occurred up to pH 10 (Smith et al., 2016). Survival up to pH 12 was possible by the formation of flocs and the production of extracellular polymeric substance (EPS; Charles et al., 2017).

It is expected that the cementitious materials used during geological disposal of Eurobitum will induce high alkaline conditions resulting in a pH above 12.5 for thousands of years and afterwards gradually drops to pH 10 (Wang et al., 2007; Valcke et al., 2022). However, several pH lowering processes (e.g., degradation of bitumen into CO_2_ or organic acids) can be present in a repository, which may result in small niches with lower pH (Valcke et al., 2022). Furthermore, at the interface of Boom Clay with the disposal gallery, the pH is not expected to be higher than pH 10.5. The alkaline plume is expected to reach the first 1–3 meters from the concrete—Boom Clay interface, while further in the Boom Clay, the pH will not change and will remain pH ~ 8.4 (Wang et al., 2007). Here, we investigate the effect of each of the expected pH conditions on microbial reduction of nitrate leaching from thermally aged non-radioactive Eurobitum during 1 year. Two microbial communities originating from different locations were used, each thriving at different pHs. On the one hand, Boom Clay borehole water from the HADES URF (underground research facility; −225 m) at SCK CEN (Mol, Belgium) is used. This microbial community thrives in a slightly alkaline environment with pH 8.4. A second community from sediment from the Harpur Hill site near Buxton (UK) is used as well. Features and processes occurring in this sediment are considered as analogous to some conditions present in a cementitious geological disposal facility. Consequently, the present microbial community is adapted to thrive in high pH conditions (Smith et al., 2016). Sodium acetate was added as known bitumen degradation product to enhance nitrate reduction rates (Mijnendonckx et al., 2020).

## Materials and methods

### Microbial inocula

#### Boom Clay borehole water

The Boom Clay formation was accessed through the HADES URF of SCK CEN (Mol, Belgium), a concrete-lined gallery at a depth of 225 meters below the surface. A detailed overview of the geology of Boom Clay is given in Vandenberghe et al. (2014) and the mineralogical composition is documented in Zeelmaekers et al. (2015). The clay contains about 20 wt % of Boom Clay pore water, which mainly consists of about 14.4 mM HCO_3_^−^ and 4.2–12.5 mM C dissolved organic carbon (Honty et al., 2022). This dissolved organic fraction has a wide molecular size spectrum and is mainly comprised of highly aromatic structures and less aromatic structures richer in carboxylic acid, phenolic and ketonic groups (Bruggeman and De Craen, 2012).

The microbial inoculum for the experiments was collected from a vertical piezometer TD-11D. The piezometer, installed in 2001, was designed to study the variability of the Boom Clay pore water composition underneath the HADES research facility. It allows pore water sampling at 12 different stratigraphic levels of the Boom Clay (clayey/silty, organic rich/poor, carbonate rich/poor). All the porous filter screens of this piezometer are made out of “Schumatherm” filters, used for its chemically inert characteristics (De Craen et al., 2019). The microbial population present in the borehole water from filter 23 (code name TD-11D-23) was determined as the most representative for the microbial community present in that piezometer (Wouters et al., 2013), hence it was selected as the microbial inoculum for the experiments carried out in this study. The inoculum was collected anoxically in a 50 ml sample cylinder, which was transferred to a septum bottle in an anaerobic glove box (99% Ar/1% H_2_). The septum bottles were stored at 4°C until sufficient inoculum was collected. To avoid differences in the initial inoculum between the different conditions, all septum bottles were combined and stirred before the start of the experiments. The chemical composition of the pore water used during this study is presented in Table 1.

**Table 1:** Composition of Boom Clay borehole water used in this study.

Component	TD-11D-23	TD-116E
pH	7.88	8.56
TIC	166	169
Alkalinity (meq/l)	15.0	15.5
TOC	142	72
SO_4_^2−^	0.84 ± 0.04	2.78 ± 0.32
Cl^−^	0.80 ± 0.05	11.4 ± 1.5
K^+^	0.22 ± 0.03	4.4 ± 0.5
Si	0.21 ± 0.02	2.86 ± 0.29
F^−^	0.16 ± 0.02	21.5 ± 1.4
Mg^2+^	0.10 ± 0.01	3.07 ± 0.31
Ca^2+^	0.07 ± 0.01	3.65 ± 0.38
Br^−^	0.01 ± 0.003	0.94 ± 0.23
Fe	0.01 ± 0.003	0.282 ± 0.035

Concentrations are in mg/l unless indicated otherwise.

#### Harpur Hill sediment

Sediment was collected from the Harpur Hill site near Buxton, United Kingdom, that had been contaminated for decades by waste from a legacy lime works. The continuous percolation of residual lime (CaO) within the lime waste deposited at the site, by rainwater and shallow groundwater, has led to the development of an alkaline, calcium hydroxide dominated leachate with a pH of about 12.5 and the formation of an alkaline ‘lagoon’ of calcium carbonate tufa precipitate. However, the pH of the fluids at the site varies drastically, and is influenced by factors such as rainfall (Smith et al., 2016). Sediments comprise high calcium and silicate concentrations and are seen as analogous to some conditions present in a cementitious geological disposal facility.

### Characteristics of the bitumen samples

The non-radioactive Eurobitum used in this study originates from a drum that was produced during the non-radioactive start-up phase of the Belgian reprocessing facility Eurochemic/Belgoprocess. It contains the reference salt composition and has been used previously to assess the leaching of soluble salts from Eurobitum under free or restricted swelling conditions. The production method and composition of this non-radioactive Eurobitum are similar to that of radioactive Eurobitum, except for the presence of the radionuclides. This reference Eurobitum is characterized by a density of 1.31 g/ml and it is composed of 0.39% water and 38.9% salts of which 28.5% is NaNO_3_ (Mariën et al., 2013; Valcke et al., 2022). Thermally aged Eurobitum was prepared by heating crushed non-radioactive reference Eurobitum 0–2 CR15/16 at 180 to 190°C and subsequently mixing them with a manually manipulated electrically driven stirrer for 8 h to maximize the exposure of the bulk of the material to air. Afterwards, the aged Eurobitum was cooled down slowly to ambient temperature. The sample was stirred occasionally to reduce air bubbles formed at the bottom of the sample in the initial stage of the cooling process (Valcke et al., 2009). From the pieces of this thermally aged Eurobitum, cylindrical specimens were sampled using a sampling tube designed at SCK CEN with an inner diameter of 52 mm. These specimens were sliced in cylindrical samples with a height of 10 mm (Valcke et al., 2009) and were further cut in cubes with dimensions of *ca.* 7 mm × 7 mm × 10 mm. All cutting equipment were disinfected with 70 vol% ethanol to work as sterile as possible.

### Experimental set-up

All preparations were performed in an anaerobic glove box with a manually controlled atmosphere of 99% argon and 1% hydrogen to obtain anoxic conditions.

To mimic expected pH conditions at different locations in the disposal gallery located in Boom Clay, media with three different initial pH values were prepared, namely pH 9, pH 10.5 and pH 12 (Supplementary Figure 1). An initial pH 9 was acquired by filter sterilizing (0.22 μm) and autoclaving natural Boom Clay borehole water from piezometer TD-116E, during which some degassing occurred. The piezometer is placed horizontally in the Boom Clay, and is located in the Test Drift part of the HADES URF, at ring 116, pointing towards the east. It is entirely made of stainless steel and contains in total four large surface filter screens made from high porosity seamless filter tube made by “Krebsöge,” quality: SIKA R5, material: 1.4404 (AISI 316l/B), pore size distribution: 7 to 16 μm. This piezometer was designed in 1999 to provide sufficient quantities of representative Boom Clay pore water as feed and reference material for laboratory experiments (De Craen et al., 2019). The specific chemical composition of the pore water used in this study is presented in Table 1.

To acquire a representative and stable pH of 10.5, “Old Cement Water” (OCW) was mixed with Boom Clay borehole water. OCW is a solution with pH 11.7, comprising of 3.45 mM NaOH, 0.78 mM Ca(OH)_2,_ 0.02 mM CaCO_3_ and 0.04 mM Na_2_SO_4_. It is representative for the highly alkaline pore water in the cementitious materials of the current design of the Eurobitum disposal facility, after dissolution and leaching of portlandite, i.e., when the pH of the cement pore water will be regulated by the incongruent dissolution of C-S-H phases [degradation stage III, Valcke et al. (2022)]. Mixing 30% water from piezometer TD-116E with 70% OCW water resulted in the medium with an initial pH 10.5.

The medium with an initial pH 12.5 was prepared after saturation of water from piezometer TD-116E with Ordinary Portlandite Cement (OPC) CEM-1. More in detail, 10 polystyrene culture flasks of 75 cm^2^, were filled with 50 g OPC and 100 ml Boom Clay borehole water. The supernatant was transferred to 50 ml falcon tubes and centrifuged for 5 min at 10000 g after 1 week. The supernatant was collected and returned immediately to an anaerobic atmosphere to minimize the contact time with air. After deoxygenation of all solutions, 50% of the OPC equilibrated Boom Clay borehole water was mixed with 30% OCW water and 20% Boom Clay pore water to obtain a final pH of 12.5.

None of the media contained visible precipitates. Each medium was supplemented with 15 mM sodium acetate. Ninety ml of each medium was inoculated with 10 ml Boom Clay borehole water (filter TD-11D-23), with 2% Harpur Hill sediment or with 10 ml filter sterilized and autoclaved Boom Clay borehole water (TD-116E), serving as the abiotic control. Three blocks of thermally aged non-radioactive Eurobitum with a total weight varying from 2.75 to 2.86 g were added to all test conditions. All test conditions were performed in triplicate in 250 ml glycol-modified polyethylene terephthalate (PETG) Erlenmeyer flasks. The Erlenmeyer flasks were closed to avoid evaporation and incubated at room temperature in an anaerobic glove box (99% Ar/1% H_2_) to maintain anoxic conditions throughout the experiment. 0.2 mM Na_2_HPO_4_ was added to the samples at day 356.

The evolution of pH, nitrate, nitrite, acetate and total microbial cells was monitored by taking subsamples of 2 ml at the start of the experiment, after day 5, day 8, day 14, day 21, day 28, day 35, day 78, day 110, day 356 and day 359.

### Chemical analysis

Nitrate, nitrite and acetate were measured with a Dionex ion chromatography system equipped with a Dionex IonPac AS11-HC anion exchange column, a Dionex IonPac AS11-GC guard column and conductivity detector. Before measuring, the samples were diluted 40 × and filtered using 0.45 μm Acrodisc^®^ PSF syringe filters (VWR International, Belgium). The eluent gradient program was 1 mM sodium hydroxide for 8 min, increasing to 15 mM sodium hydroxide during the following 10 min, increasing to 30 mM sodium hydroxide in the following 10 min, in the next 10 min increasing to 60 mM sodium hydroxide and finally hold for 2 min at 60 mM sodium hydroxide. The chromatograms were collected and processed with the Chromeleon version 6.5 software. The measurement uncertainties depended on the dilution factor, detection limit and concentration of the respective element but in general typical values were 10%.

Nitrate reduction rates were calculated by subtracting the average nitrate concentration measured in the abiotic samples from the nitrate concentration in each biotic test and dividing this by time. Nitrite production rates are equal to the observed nitrite concentrations, divided by time as no nitrite was expected nor observed in the abiotic conditions. The dissolved nitrogen mass balance was calculated as the sum of the concentrations of N–NO_3_^−^ and N–NO_2_^−^.

### Cultivation

To investigate possible resuscitation of the cultures exposed to pH 12.5 for 356 days, two replicates were diluted 1/5 in fresh medium with pH 8.5 and pH 10.5. pH 8.5 was obtained by filter sterilizing and autoclaving Boom Clay borehole water of filter TD-116E supplemented with 15 mM NaHCO_3_, 1.5 mM Na_2_HCO_3,_ 20 mM NaNO_3_, 5 mM NaCH_3_COO and 0.2 mM NaH_2_PO_4_. Medium with pH 10.5 was obtained by mixing 30% sterilized water from piezometer TD-116E supplemented with 15 mM NaHCO_3_, 1.5 mM Na_2_HCO_3_ and 70% OCW. To this mixture, 20 mM NaNO_3_, 5 mM NaCH_3_COO and 0.2 mM NaH_2_PO_4_ was added to provide nutrients for bacterial growth. Samples were incubated at room temperature in the anaerobic glove box. Total cell count was measured after 7 days.

### Total cell count

Flow cytometry was used to count the total amount of microbial cells present in the samples. To this end, samples were diluted in 0.22 μm filter sterilized Evian potable water. Double stranded DNA was stained with SYBR® Green I (10 000 x concentrate in 0.22 μm filtered dimethyl sulfoxide; ThermoFisher Scientific, Belgium; final concentration of 1 × concentrate) and incubated in the dark for 20 min at 37°C. Flow cytometry was performed using a C6 AccuriTM flow cytometer with autosampler (BD Biosciences, Belgium), which was equipped with four fluorescence detectors (530/30 nm, 585/40 nm, > 670 nm and 675/25 nm), two scatter detectors and a 20-mW 488-nm laser. The flow cytometer was operated with Milli-Q filtered water (Merck Millipore, Belgium) as sheath fluid. Samples were analyzed in a fixed volume mode of 50 μl and the threshold was fixed on the green fluorescence (FL1-H at 1000). Flow cytometry data were extracted under the Flow Cytometry Standard format, imported in R (v.3.6.0) and analyzed with the Phenoflow package developed by Props et al. (2016).

### Scanning electron microscopy

One of the non-radioactive Eurobitum cubes present in the tests was used to perform SEM analysis. The piece of non-radioactive Eurobitum was washed with 10 mM MgSO_4_ for 10 min to remove residual microbial cells that were not attached to the Eurobitum. The Eurobitum samples were fixed overnight with solution containing 0.3 M gluteraldehyde and 0.132 M sodium cacodylate. Afterwards, excess gluteraldehyde was removed by washing the filter with sodium cacodylate solution (0.150 M) twice. Subsequently, cells were dehydrated using an ascending graded series of ethanol solutions (30, 50, 70, 90, 95% v/v), followed by a final solution of 100% ethanol, which was replaced twice (minimum 10 min between each solution). Drying of the Eurobitum samples was performed twice with hexamethyldisilazane for 2 min, followed by air drying for at least 1 h. Finally, a small piece was sliced with a sterile razor blade and mounted on a copper stub using carbon conducting tape and nail polish. These sample slices were sputter-coated with gold (20 nm) in one cycli of 200 s (4 mbar Argon, 50 mA, 1 kV; Scancoat Six, BOC Edwards B.V., Dongen, Netherlands). SEM analysis was performed on a Phenom ProX (Phenom-World, Netherlands), equipped with a backscatter electron detector at a working distance of 20 mm and a 10 or 15 kV acceleration.

### DNA extraction

DNA extraction was based on Povedano-Priego et al. (2021) with small modifications. In brief, 1 ml of the sampled solution of day 110 and 50 ml of the samples of day 356 and after the addition of phosphate were filtered (0.22 μm Supor^®^ PES, Pall Corporation, Belgium) to collect all microorganisms. In addition, DNA of 50 ml of Boom Clay borehole water and of 1 gram Harpur Hill sediment used as inoculum was extracted. The filters and soil sample were placed in a Lysis matrix E tube (MP Biomedicals, Netherlands) and suspended by vortexing in 400 μl Na_2_HPO_4_ (0.12 M, pH 8.0). Afterwards, 600 μl lysis buffer containing 100 mM Tris–HCl (pH 8.0), 100 mM EDTA (pH 8.0), 100 mM NaCl, 1% PVP and 2% SDS were added to each tube followed by the addition of 24 μl freshly made lysozyme (10 mg/ml) and 2.5 μl proteinase K (20 mg/ml). Then, mechanical lysis was performed by vortexing the tubes 10 min at maximum speed with a Vortex adapter cat 13,000-V1 (Qiagen, Netherlands). The samples were incubated at two different temperatures: first 30 min at 37°C followed by 45 min at 60°C. Next, the samples were centrifuged for 5 min at 14000 g at room temperature and the supernatant was collected in a new 15 ml Falcon tube (VWR, Belgium). The pellets were again suspended in 1 ml lysis buffer and the mechanical lysis by vortexing for 10 min at maximum speed was repeated. The samples were centrifuged for 5 min at 14000 g and the supernatant was again transferred to the Falcon tube. To all samples, one volume of phenol:chloroform:isoamylalcohol (25:24:1 v/v) was added and the tubes were mixed gently by inverting the tubes. After centrifugation at 1500 g for 10 min at 4°C, the upper (aqueous) phase was transferred to a new tube and washed by adding one volume of chloroform:isoamylalcohol (1:1 *v*/*v*). Tubes were again centrifuged at 1500 g for 10 min at 4°C and the supernatant was transferred to a new tube. Afterwards, DNA was precipitated by adding 2.5 volume of 100% ethanol and 1/10 volume of 3 M sodium acetate (pH 5) and overnight incubation at −20°C. Then, the sample was centrifuged 30 min at 10000 g, the pellet was washed with 5 ml of a 70% ethanol solution and centrifuged for 15 min at 10000 g. After drying the pellet to remove all ethanol, the pellet was dissolved in 500 μl milli-Q water and stored overnight at 4°C. Subsequently, the sample was applied on a 100 kDa amicon filter unit (Merck, Belgium) and centrifuged for 10 min at 14000 g. The DNA was washed twice with 500 μl milli-Q water. Finally, DNA was eluted by centrifugation for 2 min at 1500 g.

### 16S rRNA amplicon sequencing

High-throughput amplicon sequencing of the V3–V4 hypervariable region of the 16S rRNA gene was performed with the Illumina MiSeq platform according to the manufacturer guidelines at BaseClear B.V (Netherlands). DNA sequencing data were processed using the OCToPUS pipeline (Mysara et al., 2017), which consists of the following steps: quality filtering using HMMER, merging reads using the make.contigs command from the open source software package mothur (v.1.39.1), alignment and filtering of the merged reads following the standard operating procedure (SOP) as described by the authors of the mothur software, error correction using IPED (Mysara et al., 2016) chimera identification using CATCh (Mysara et al., 2015) and OTU (Operational Taxonomic Unit) clustering using UPARSE (v7.0) using a 97% cut-off (Edgar, 2013). The datasets generated and analyzed during the current study are available in the NCBI Sequence Read Archive (SRA) repository (PRJNA847218).

### Statistical analysis

Nitrate leaching was compared between the different sterile conditions through a two-way ANOVA using the mixed-effect model with the Geisser–Greenhouse correction followed by a Tukey’s multiple comparisons test. Normality was checked with a QQplot. The 16S rRNA amplicon sequencing data were analyzed in R version 4.1.0 with the R package phyloseq (McMurdie and Holmes, 2013; R Core Team, 2017). Subsampling was performed based on the lowest amount of reads obtained over the 19 different samples, i.e., a coverage of 18,549 reads. Rarefaction curves indicate that this level of subsampling adequately represented the bacterial diversity in the samples (Supplementary Figure 2). Alpha diversity was determined using Chao, Shannon Diversity and Inverse Simpson index using the command “estimate_richness” in the package phyloseq. The β-diversity was calculated by non-metric multidimensional scaling (NMDS) with weighted UniFrac distances with the command “ordinate.” Afterwards, a distance matrix of these data was calculated with the command “distance.” This distance matrix was used to perform a permutation test for homogeneity of multivariate dispersions using the command “betadisper” in the package vegan (Oksanen et al., 2020). Permutational Multivariate Analysis of Variance (PERMANOVA) using the “adonis” at 999 permutations and *α* = 0.05 were performed testing the differences between both communities in general and between different pH conditions. Pairwise multilevel comparison was performed with the R package pairwiseAdonis with FDR (False Discovery Rate) correction to the *p*-values. Significantly different OTUs among the groups were examined with the R package indicspecies using the “multipatt” command. Correlation indices were determined with the function “r.g,” which includes a correction for unequal group sizes (Cáceres and Legendre, 2009).

## Results and discussion

### pH evolution

**Figure 1:**
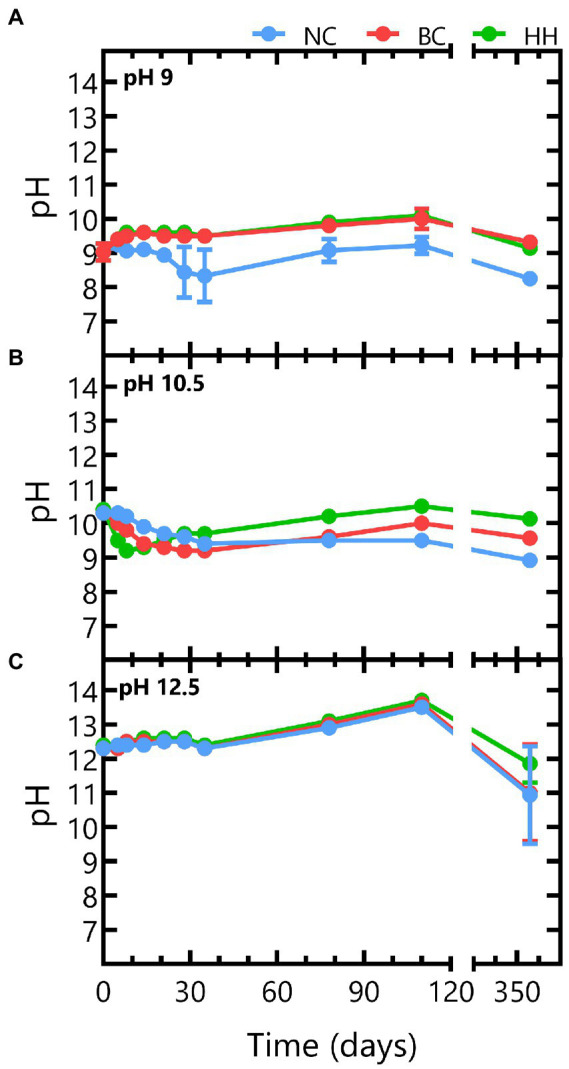
pH evolution for abiotic tests (blue) and for biotic tests with Boom Clay borehole water (red) and Harpur Hill sediment (green) in conditions with initial **(A)** pH 9, **(B)** pH 10.5, and **(C)** 12.5. Results are presented as the average and standard deviation of three replicates, except for day 356 and abiotic samples of pH 9 and 10.5 where the result of two replicates is shown.

Batch experiments with different initial pH were setup and monitored during 356 days to assess microbial nitrate reduction at alkaline pH. Abiotic control tests with an initial pH 9 remained constant up to day 110, after which the pH decreased to pH 8.25 (Figure 1A). In the biotic tests, the pH increased rapidly to pH 9.5 (within a 1 week) and increased further to pH 10 up to day 110. As this evolution is notably different from the evolution in the abiotic control tests, it is likely caused by microbial activity. After 356 days, the pH had decreased again to pH 9.3 in the tests with Boom Clay borehole water and to pH 9.1 for the tests with Harpur Hill sediment. A similar pH decrease was observed for the abiotic test (Figure 1A), which suggests that it is not linked to microbial activity.

In the test conditions with an initial pH 10.5, a gradual decrease in pH was observed under abiotic conditions. After ~ 1 month, the pH had decreased to 9.6 and remained stable afterwards up to day 110. This pH decrease was more pronounced in the biotic conditions and more prominent for the Harpur Hill sediment, where it was already observed after 1 week, while for the Boom Clay borehole water a similar decrease up to pH 9.2 was only visible after *ca.* 1 month. After the observed pH decrease, pH gradually increased again to pH 10.5 ± 0.1 for the Harpur Hill sediment and to pH 10 ± 0.2 for the Boom Clay borehole water at day 110. Again, this pH increase was more pronounced and occurred faster for the Harpur Hill sediment than for the Boom Clay borehole water (Figure 1B).

All tests with an initial pH 12.5 showed a similar pH profile and pH remained above 12 up to 110 days. More variation between the replicates was observed at day 356, as the pH decreased to ~12 in one replicate but to pH 10 in the other replicate for the abiotic control and the Boom Clay borehole water community and to pH 11.4 for the Harpur Hill sediment (Figure 1C).

Overall, in all abiotic tests, a pH decrease can be observed on the long term. This may be caused by some uptake of CO_2_ from the atmosphere (in the glove box) or by acidification due to bitumen degradation. Furthermore, leaching of Ca^2+^ from the Eurobitum samples may have resulted in the formation and precipitation of CaCO_3_, thereby releasing protons. Leaching of Ca^2+^ and SO_4_^−2^ from Eurobitum was however not followed up in detail in our experiments. Nevertheless, it is expected that leaching is diffusion controlled but suppressed in a cementitious environment by the low solubility of CaSO_4_ (Sneyers and Van Iseghem, 1998). The limiting Ca^2+^ concentration (leached and originally added to the medium) could explain the initial stable pH observed in the abiotic tests starting at pH 12.5.

### NaNO_3_ leaching from thermally aged bitumen under free swelling conditions at different pH

**Figure 2:**
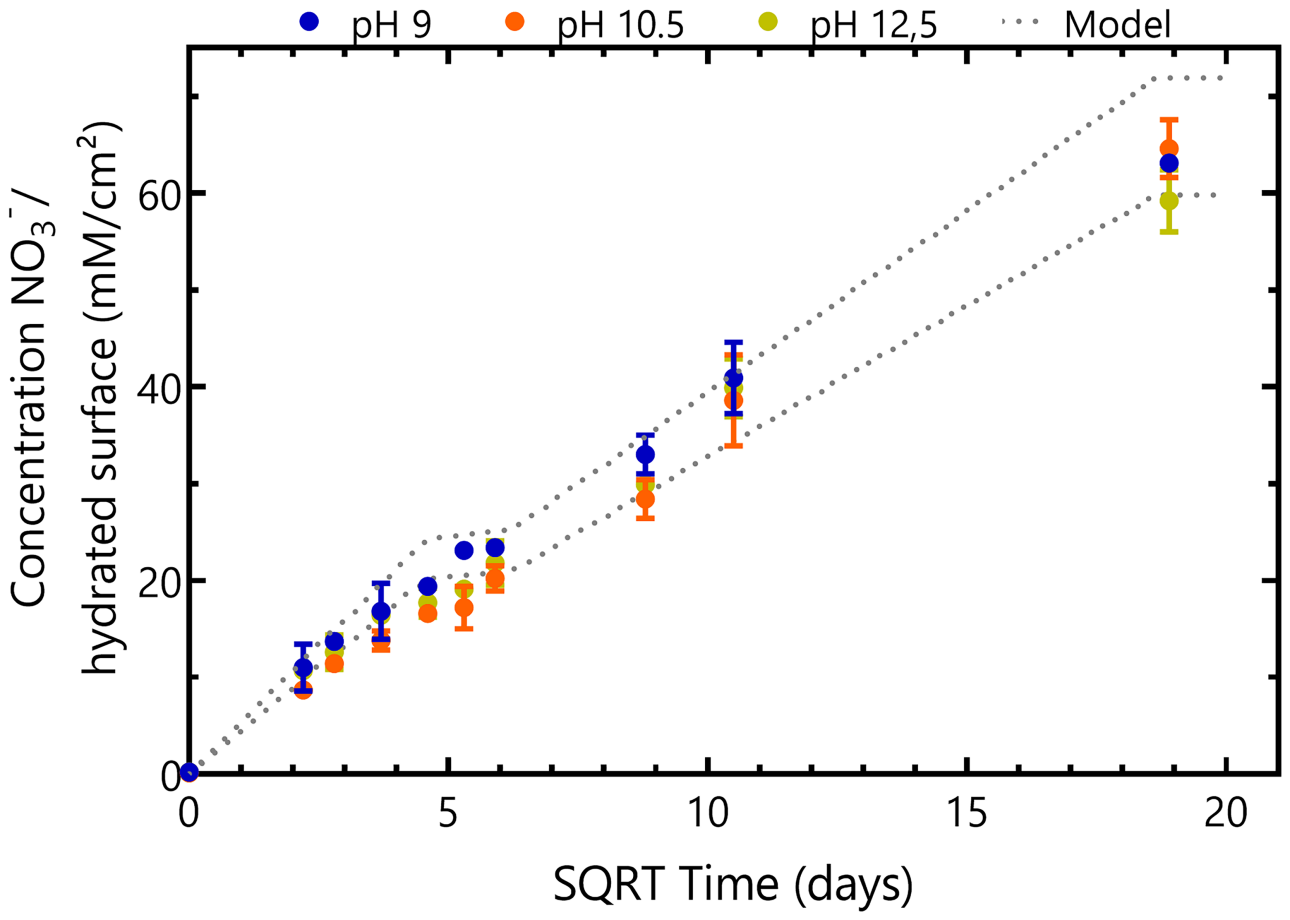
Time evolution of NaNO_3_ concentrations in abiotic samples leached from thermally aged non-radioactive bituminized waste under free swelling conditions. Concentrations of conditions with initial pH 9 are presented in blue, conditions with initial pH 10.5 are shown in yellow and orange represents conditions with initial pH 12.5. Values represent the average and standard deviation of two replicates for pH 9 and pH 10.5 and three replicates for pH 12.5. The dotted line represents the maximum expected leaching rates where the lowest line shows the calculated leaching rates for the Eurobitum cube containing the least amount of nitrate and the highest line represents the Eurobitum cube containing the most NaNO_3_. After 348 days (the start of the second plateau), all nitrate is expected to be leached from the Eurobitum cubes.

NaNO_3_ leaching from Eurobitum is pH independent as the same leaching profile was observed in all abiotic conditions (Figure 2). NaNO_3_ is leaching fast the first 28 days, from pores close to the surface. The leaching process proceeds much slower between day 28 and day 35 as it is diffusion-limited (Figure 2). Afterwards, there is again a re-increase in the leaching rate, which can be attributed to the formation of interconnecting pores and (micro)cracks in the bitumen matrix in response to its increasing deformation (Bleyen et al., 2016a). Almost all nitrate is leached in all conditions after 356 days. Overall, this leaching behavior and the total amount of NaNO_3_ leached from the samples is in agreement with predicted leaching behavior based on the amount of non-radioactive Eurobitum present in the test solutions and on the leaching rates (normalized to the initial contact surface) observed by Bleyen et al. (2016a; Figure 2). In the latter study, water uptake and leaching of salts from non-radioactive Eurobitum under free swelling conditions and in 0.1 M KOH (pH 13) was investigated.

### Impact of pH On nitrate reduction

**Figure 3:**
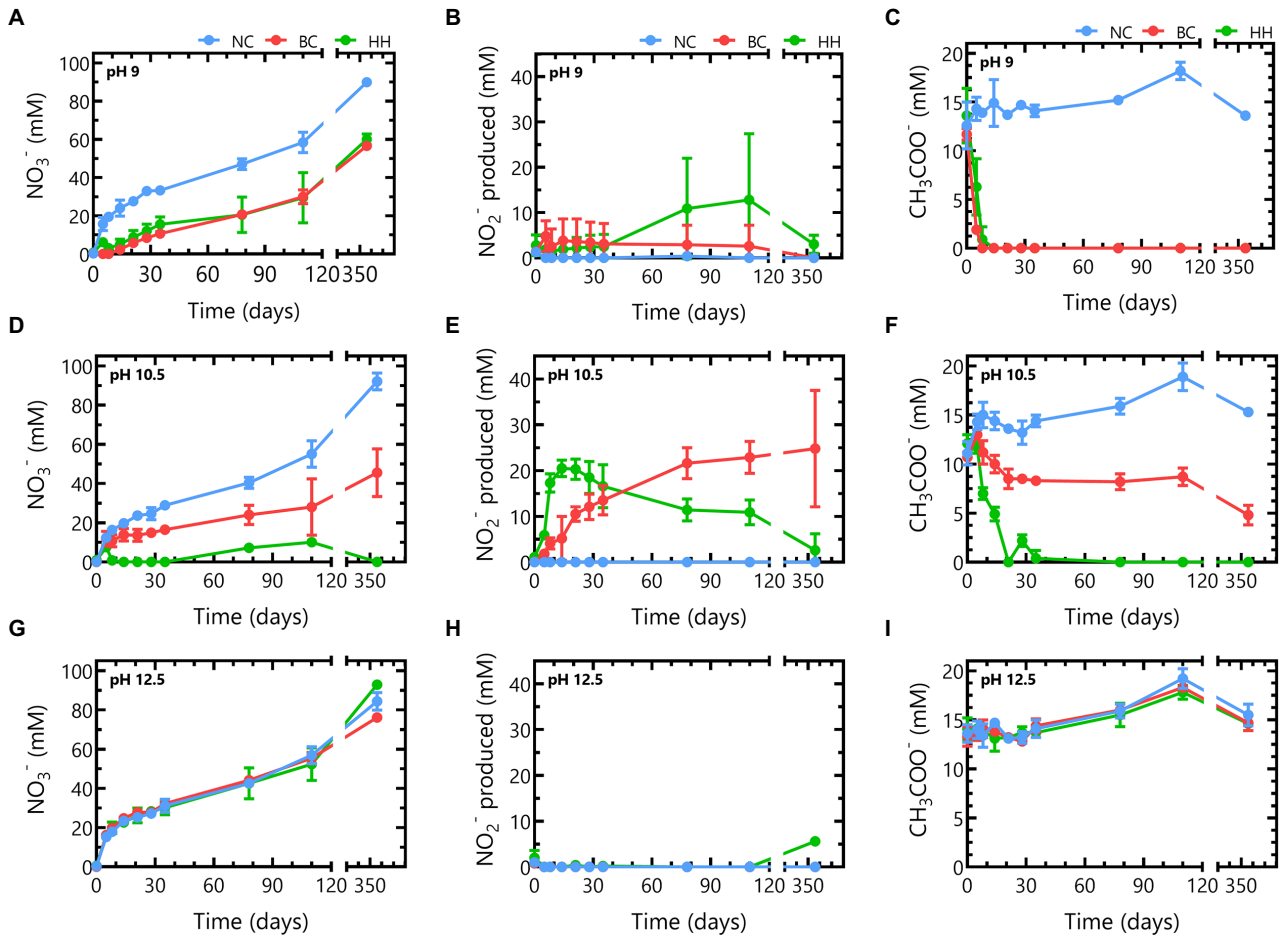
Nitrate (**A,D,G**), nitrite (**B,E,H**) and acetate (**C,F,I**) concentrations in abiotic samples (blue), samples inoculated with Boom Clay borehole water (red) or samples inoculated with Harpur Hill sediment (green) during 356 days of incubation in the presence of thermally aged non-radioactive Eurobitum at pH 9 (**A-C**), pH 10.5 (**D-F**) and pH 12.5 (**G-I**). Values represent the average and standard deviation of three replicates, except for day 356 and abiotic samples of pH 9 and pH 10.5 where the result of two replicates is shown.

Nitrate, nitrite and acetate concentrations were monitored during 356 days to investigate the impact of pH on nitrate reduction rates of the microbial communities from Boom Clay borehole water and of the Harpur Hill sediment (Figure 3).

The Boom Clay borehole water community reduced all nitrate leached from the Eurobitum during the first 8 days at pH 9. A maximum nitrate reduction rate of ~ 2.9 mM NO_3_^−^/day was observed during the first 5 days (Figure 3A). The lag phase for the Harpur Hill sediment seemed to be longer as the nitrate reduction rate was only ~ 1.6 ± 0.4 mM NO_3_^−^/day during the first 5 days, which resulted in a small build-up of nitrate, leaching from the Eurobitum. The maximum nitrate reduction rate, ~ 2.2 ± 0.4 mM NO_3_^−^/day, was observed between 5 and 8 days and was only slightly lower compared to that of the Boom Clay borehole water. A similar amount of nitrate was reduced by both communities after 356 days (Figure 3A). The highest nitrate reduction rates occurred for both communities concomitantly with a rapid decrease in acetate (Figure 3C), indicating that acetate is used as electron donor for the observed nitrate reduction. Nitrite production seems similar for both communities up to 35 days. Afterwards, nitrite concentrations remain constant for the Boom Clay borehole water, while in one of the replicates of the Harpur Hill sediment, an increase was observed (Figure 3B). Although the production of nitrogen gases was not monitored, it can be assumed that the reduction of nitrate to nitrous oxide is not a dominating reaction. Thus, a considerable accumulation of nitrous oxide in the samples is not expected to occur. Consequently, nitrogen gas would be the end product of denitrification (Bleyen et al., 2016b). This assumption taken into account, the following reactions could occur in the presence of acetate [Eqs. (1) and (2) derived from Madigan (2012)]:


(1) $$\begin{array}{c} {\mathrm{CH}}_{3}{\mathrm{COO}}^{–}+4\;{{\mathrm{NO}}_{3}}^{–}\to 4\;{{\mathrm{NO}}_{2}}^{–}+2\;{{\mathrm{HCO}}_{3}}^{–}+H^{+}\;({\text{∆G0}}^{\prime }\\ \left(\mathrm{pH}\;7\right)=–69\;\mathrm{kJ}/\mathrm{mol}\;e^{–}) \end{array}$$

(2) $$\begin{array}{c} 5\;{\mathrm{CH}}_{3}{\mathrm{COO}}^{–}+8\;{{\mathrm{NO}}_{3}}^{–}+3\;H^{+}\to 4\;N_{2}+10\;{{\mathrm{HCO}}_{3}}^{–}+4\;H_{2}O({\text{∆G0}}^{\prime }\\ \left(\mathrm{pH}\;7\right)=–101\;\mathrm{kJ}/\mathrm{mol}\;e^{–}) \end{array}$$

**Figure 4:**
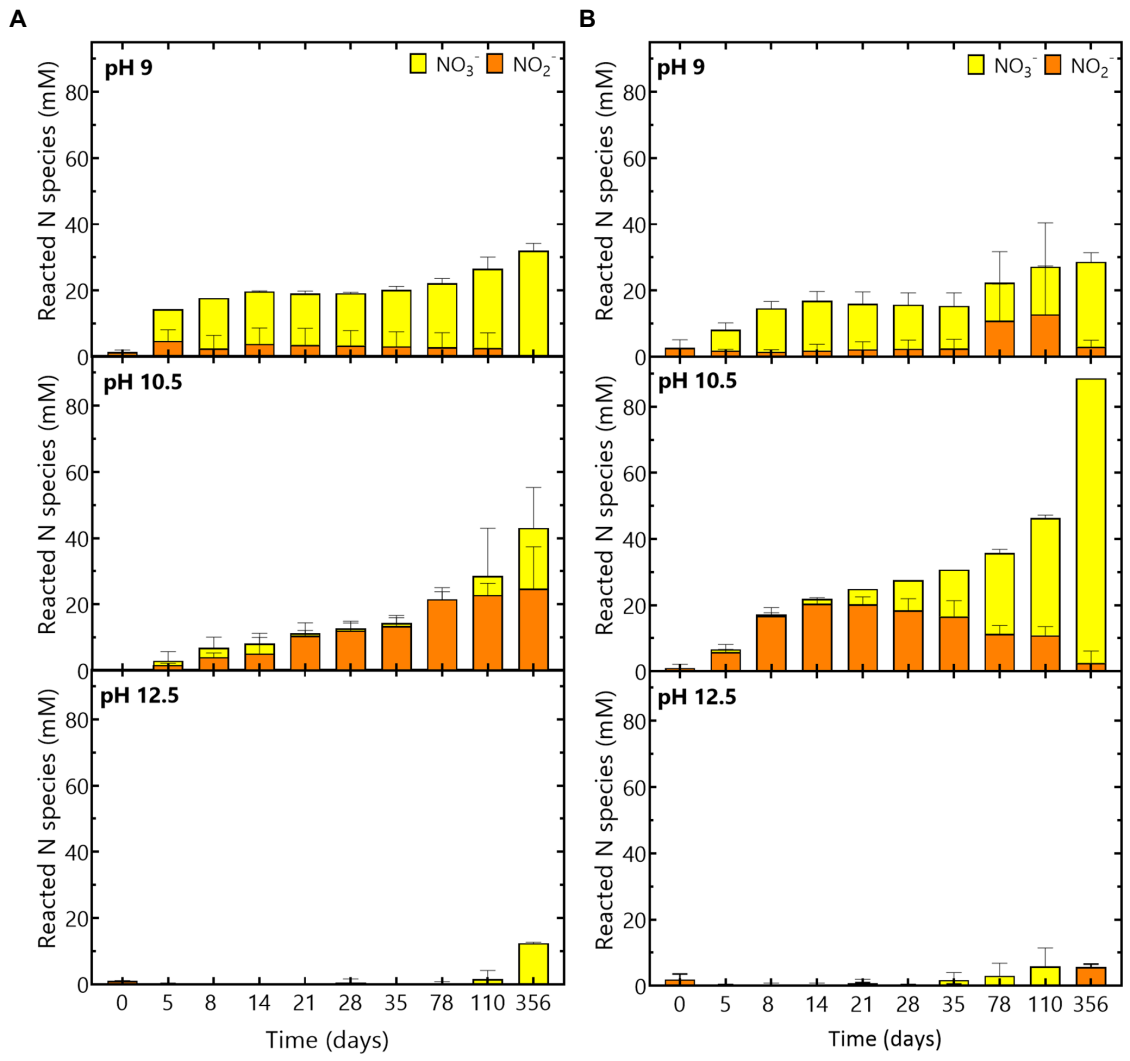
Evolution of the reacted N species (NO_3_^−^ and NO_2_^−^) during the experiment at pH 9, pH 10.5 and pH 12.5 for **(A)** the Boom Clay borehole microbial community and **(B)** the Harpur Hill sediment. NO_3_^−^ concentrations are given in yellow and NO_2_^−^ concentrations are shown in orange. Values represent the average and standard deviation of three replicates except for day 356 and abiotic samples of pH 9 and pH 10.5 where the result of two replicates is shown.

Based on the reacted N species (Figure 4A) and the increase in pH (Figure 1), it seems that reaction (2) is the most dominant reaction. The remainder of the acetate (*ca.* 37% for the Harpur Hill sediment and *ca* 22% for the Boom Clay borehole water community) could have been used as carbon source, which resulted in an increase of the total cell number during acetate consumption (Supplementary Figure 3) and is in good agreement with previously reported data (Clement et al., 1997; André et al., 2011; Parmentier et al., 2014). When all acetate was consumed, nitrate reduction was limited up to day 35 for the Harpur Hill sediment and up to day 78 for the Boom Clay borehole water (Figures 3A, 4). Nitrate was again removed afterwards albeit at a lower rate. Although it cannot be excluded that the 1% H_2_ present in the anaerobic glove box that putatively leaks in the flasks could be used as electron donor, a previous study suggested that the organic material leached from the Eurobitum was rather used to fuel the nitrate reduction (Mijnendonckx et al., 2020).

Different observations could be made at pH 10.5. The Boom Clay borehole water microbial community reduced only 0.6 ± 0.5 mM NO_3_^−^/day during the first 5 days. The maximum rate was observed between day 5 and day 8 and was only 1.3 ± 0.2 mM NO_3_^−^/day (Figure 3B). Furthermore, the Boom Clay borehole water community did not oxidize all acetate (Figure 3B) and the total cell concentration did not show the same increase as at pH 9 (Supplementary Figure 3). This could indicate that pH 10.5 would hamper nitrate reduction by the Boom Clay borehole water community. However, the medium with pH 10.5 contains only 30% natural Boom Clay borehole water, which serves as the phosphate source for the microbial community. Previously, it has been shown that phosphate rapidly becomes limiting for acetate dependent nitrate reduction in batch experiments (Mijnendonckx et al., 2020). Phosphate in Boom Clay is bioavailable in the mineral fraction such as apatite (0.1–0.2 wt %; Honty et al., 2022), hence it is not assumed to be a limiting factor for microbial nitrate reduction in a radioactive waste repository. To test if phosphate was a limiting factor in the present conditions, 0.2 mM Na_2_HPO_4_ was added to the samples at day 356. This resulted in a rapid consumption of acetate after 3 days, a concomitant increase in nitrate reduction, nitrite production and total cell count (Supplementary Figure 4). This indicates that not pH but rather phosphate was limiting in these conditions. No effect of the phosphate addition was observed for the Boom Clay borehole water community at pH 9 where all acetate was already consumed (Supplementary Figure 4), confirming that phosphate is not the rate-limiting factor for nitrate reduction with organics from the clay water or Eurobitum as electron donor. This is in agreement with a previous study on nitrate reduction in Boom Clay pore water, where phosphate only affected the nitrate reduction rates in the presence of acetate and not when dissolved organics in the pore water were the only electron donor (Bleyen et al., 2018a,b).

On the other hand, the maximum nitrate reduction for the Harpur Hill community at pH 10.5 was 3.4 ± 0.8 mM NO_3_^−^ /day, which was higher than that at pH 9. The Harpur Hill community reduced almost three times as much nitrate after 356 days than at pH 9 (Figure 3D). This indicates that the Harpur Hill sediment community is more efficient at pH 10.5 compared to pH 9. Nitrate could only be detected at day 5, day 78 and day 110. At day 5, the microbial community was probably still adapting as also almost no acetate was consumed (Figure 3F). Afterwards, acetate was oxidized, while nitrate was reduced and nitrite rapidly accumulated (Figure 3). After ~ 1 month, all acetate was depleted, though nitrate reduction was continued, possibly with organics from the sediment or from chemical degradation of the Eurobitum. Also the nitrite concentration decreased gradually, indicating a slow nitrite reduction process, which may be either abiotic or biotic (Bleyen et al., 2016a,b, 2017). Putatively, the amount of soluble organic compounds leached from the Eurobitum or sediment at day 78 and at day 110 was too little to reduce all nitrate leached from the Eurobitum, resulting in the temporary accumulation of nitrate in the medium. Based on the mass balance of N species (Figure 4) and the rapid decrease of the pH in the beginning of the experiment (Figure 1), it seems that for the Harpur Hill sediment, reaction (1) was mostly occurring in the presence of acetate, while afterwards, nitrate was reduced to nitrogen gas (or ammonium), which is in agreement with the observed pH increase (Figure 1). Similarly, nitrite accumulation by the Harpur Hill sediment was also observed during the degradation of isosaccharinic acid at pH 10 (Bassil et al., 2015). However, based on the high C/N ratio in the beginning of the experiments, complete denitrification is expected to occur predominantly (Oh and Silverstein, 1999), similar to what was observed at pH 9 when acetate was still present. Moreover, nitrate concentrations are low and cannot explain the observed nitrite accumulation (Albina et al., 2019). On the other hand, it is known that pH can also influence denitrification reactions, i.e., as nitrite reduction would result in a pH increase, bacteria tend to accumulate nitrite rather than reduce it when the pH is already high (Albina et al., 2019). Increased pH can also affect the microbial community, which can also influence the number and activity of complete denitrifiers in the community. For the Boom Clay borehole water community, reaction (1) seems also more dominant based on the slow decrease in pH (Figure 1), but it is a slower process compared to that for the Harpur Hill sediment. As the mass balance of aqueous nitrogen species remains similar as the negative control (Figure 4), does not change by addition of phosphate (Supplementary Figure 4) and the pH re-increase is not considerable (compared to the one observed for Harpur Hill; Figure 1), it seems that the microbial community of the Boom Clay borehole water is unable to perform complete denitrification under these specific conditions and independently from the presence of phosphate. On the other hand, next to pH, limited phosphate concentrations could have influenced to observed reaction rates (see above).

Neither the Boom Clay borehole water community nor the Harpur Hill sediment was able to reduce nitrate or oxidize acetate at pH 12.5. Furthermore, addition of phosphate did not result in an increase in microbial activity, indicating that the high pH inhibits nitrate reduction.

### Biofilm formation on the Eurobitum cubes

**Figure 5:**
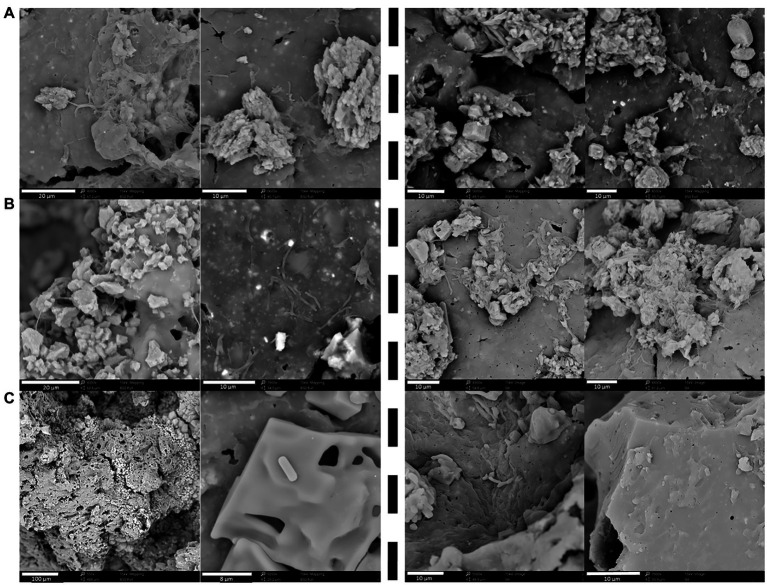
Biofilm formation on the Eurobitum cubes visualized with scanning electron microscopy (SEM) of thermally aged non-radioactive Eurobitum inoculated with Boom Clay Borehole water (left of the dotted line) or inoculated with Harpur Hill sediment (right of the dotted line) obtained after 110 days at **(A)** pH 9; **(B)** pH 10.5 and **(C)** pH 12.5.

One replicate of each condition was sacrificed after 110 days to investigate possible biofilm formation at the surface of the non-radioactive Eurobitum (Figure 5). Cells and biofilm were visible for both communities at pH 9 and pH 10.5. No biofilm was visible on the Eurobitum cubes at pH 12.5, although a few single cells were visible for the Harpur Hill sediment and putatively also for the Boom Clay borehole water. A biofilm may enhance the degradation of Eurobitum (Dasgupta et al., 2013; Flemming et al., 2016), resulting in an increased leaching of salts and organic compounds and to an increase in CO_2_ production when the organic compounds (e.g., acetate or formate) are degraded. Consequently, the formation of a biofilm could result in an underestimation of the nitrate reduction rates in our experiments.

### Impact of pH on the microbial community composition and diversity

To investigate the impact of pH on the microbial community composition, 16S rRNA amplicon sequencing was performed at day 110, at day 356 and after the addition of phosphate. Sequencing was unsuccessful for Boom Clay borehole water pH 12.5 at day 356, Harpur Hill sediment replicate 3 at pH 10.5 on day 110 and the samples of the Harpur Hill sediment at pH 12.5.

**Figure 6:**
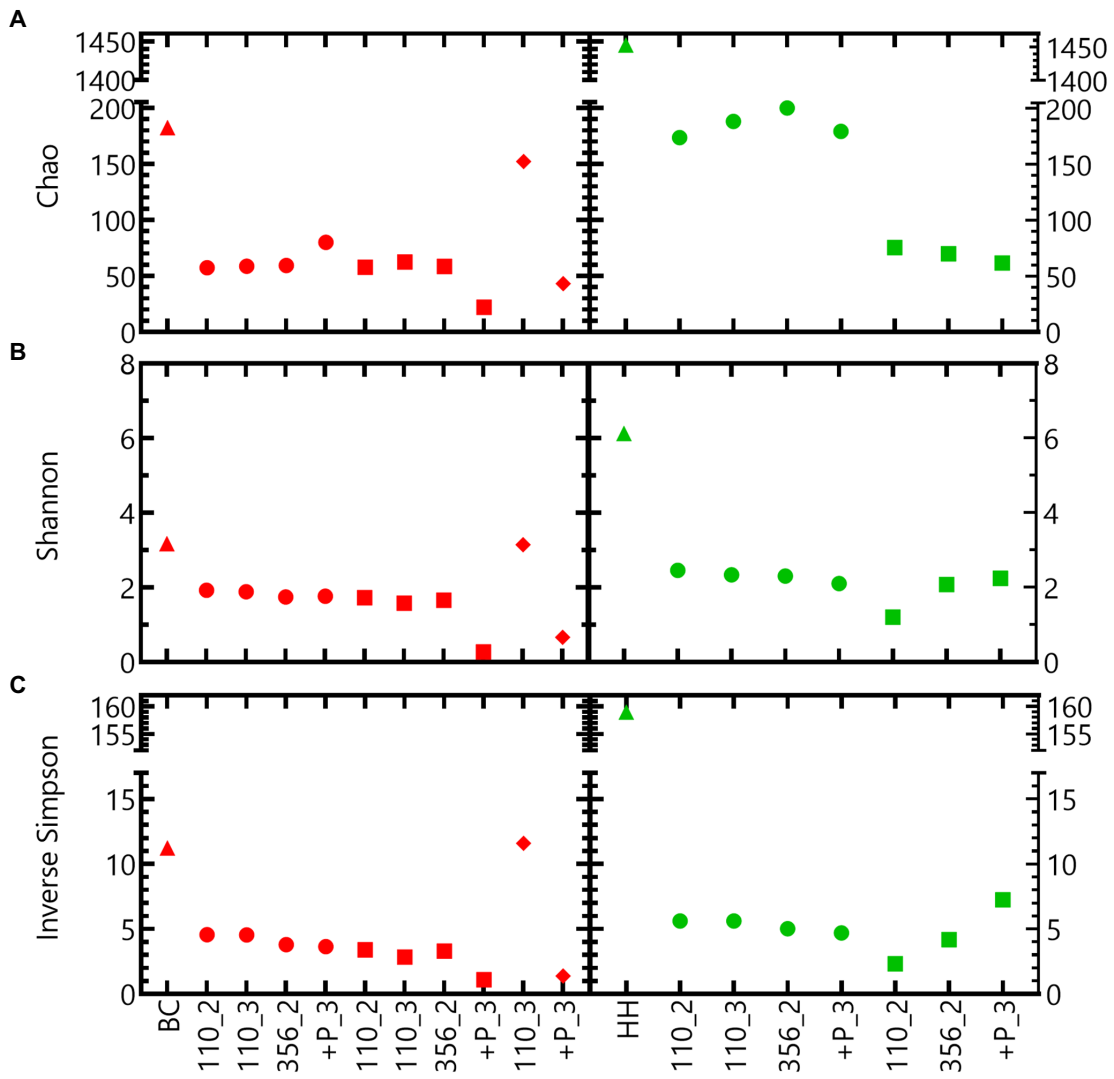
Alpha diversity indices (**(A)** Chao, **(B)** Sannon and **(C)** Inverse Simpson index) of tests inoculated with Boom Clay borehole water (red) and Harpur Hill sediment (green) at initial pH 9 (circles), pH 10.5 (squares) and pH 12.5 (diamonds). The initial community is presented as triangles.

Different alpha diversity indices indicate that the microbial community of the Harpur Hill sediment is more diverse compared to that of the Boom Clay borehole water (Figure 6). For both communities, the inoculum is more diverse compared to the samples in nitrate reducing conditions, except for Boom Clay borehole water community exposed to pH 12.5 for 110 days (Figure 6). The alpha diversity of the community in the Boom Clay borehole water exposed to pH 9 did not differ to that of pH 10.5. The addition of phosphate resulted in a lower alpha diversity at pH 10.5 and putatively also at pH 12.5. Different observations could be made for the community of the Harpur Hill sediment. The Chao index was higher in the samples exposed to pH 9 compared to those exposed to pH 10.5, which was not observed. On the other hand and values were more similar should be removed. The sentence will be: The Chao index was higher in the samples exposed to pH 9 compared to those exposed to pH 10.5, which was not observed when evenness was taken into account (Figure 6). Although we only have a limited amount of samples, these results suggest that heterotrophic nitrate reducing conditions largely reduce the richness of a microbial community. pH has an additional impact on the community richness, depending on the microbial community and its origin. A significant reduction in richness was also observed for the Harpur Hill sediment during the degradation of isosaccharinic acid in nitrate reducing conditions at pH 10 (Bassil et al., 2015).

**Figure 7:**
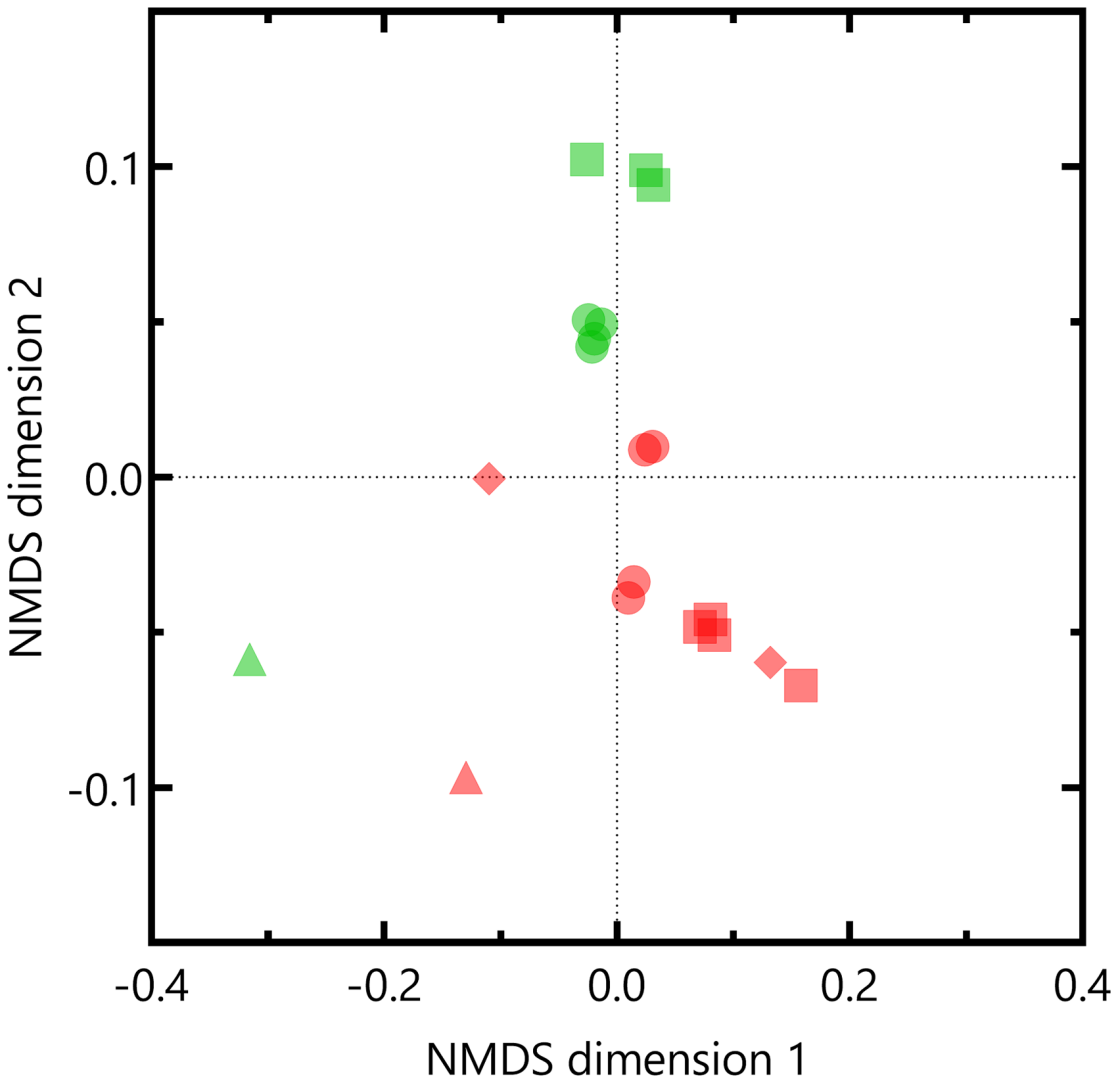
Non-metric multidimensional scaling (NMDS) based on weighted Unifrac distances of the OTUs of the bacterial community in the Boom clay borehole water (red) and Harpur Hill sediment (green) at initial pH 9 (circles), pH 10.5 (squares) and pH 12.5 (diamonds). The initial community is presented as triangles.

Nonmetric multidimensional scaling based on weighted UniFrac distances indicated that the microbial community composition of the Boom Clay borehole water was different compared to that of the Harpur Hill sediment (*p* = 0.001 according to PERMANOVA; Figure 7). Except for pH 12.5, samples within each pH condition were similar to each other as they clustered together per community. One of the Boom Clay borehole water samples exposed to pH 12.5 clustered with a sample exposed to pH 10.5 (Figure 7). Both samples were supplemented with phosphate, which could have affected the community composition as at least for pH 10.5, phosphate was shown to be a limiting factor for nitrate reduction (Supplementary Figure 4). Pairwise comparison showed significant differences (*p* < 0.05) between the Harpur Hill sediment community compositions in all conditions indicating that pH induces a shift in the microbial community composition. Note that as no data were present for the Harpur Hill sediment exposed to pH 12.5, only samples of pH 9 and pH 10.5 were taken into account to investigate the effect of pH.

**Figure 8:**
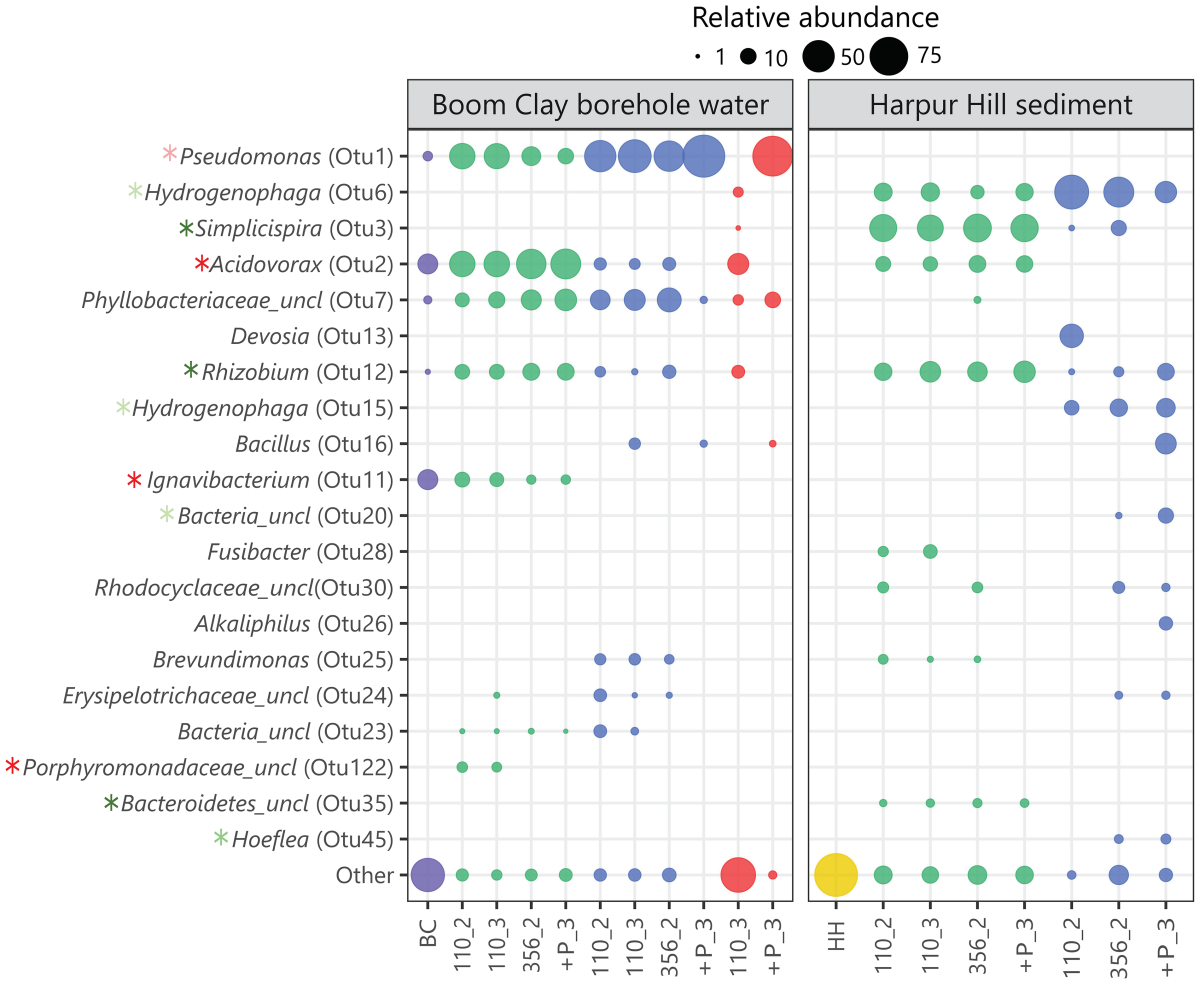
Bubble plot showing the relative abundance of the 20 most varying OTUs. The initial Boom Clay borehole water community is represented in purple, the Harpur Hill sediment is shown in yellow, conditions with initial pH 9 are indicated in green, with initial pH 10.5 in blue and with initial pH 12.5 are shown in red. OTUs significantly associated with Boom Clay borehole water at initial pH 9 or pH 10.5 are indicated with a dark red and light red asterisk, respectively. OTUs significantly associated with Harpur Hill sediment at initial pH 9 or 10.5 are indicated with a dark green and light green asterisk, respectively. Sample names are encoded as day_replicate; + P indicates phosphate addition.

Seventy eight out of 1732 OTUs that were identified in total among the groups were significantly associated with a single group. In particular, 18 and 4 OTUs were significantly associated with the Boom Clay borehole water samples exposed to pH 9 and 10.5, respectively and 49 and 7 were significantly associated with the Harpur Hill sediment exposed to pH 9 and 10.5, respectively (Supplementary Table 1). The relative abundance of most of these OTUs was very low, except for a few of each group (Figure 8; Supplementary Table 1). OTU2, member of the *Acidovorax* genus, OTU11, member of the *Ignavibacterium* and OTU122, an unclassified member of the *Porphyromonodaceae* were significantly correlated with the Boom Clay borehole water samples with initial pH 9 with OTU122 and OTU11 only present in the Boom Clay borehole water samples (Figure 8; Supplementary Table 1). There are only a low amount of cultivated representatives of the genus *Ignavibacterium* so it is not well-known. Nevertheless, it is identified during microbial community studies at distinct places, including Boom Clay borehole water (Gavrilov et al., 2017; Mijnendonckx et al., 2019). The pH range for growth of type strain *Ignavibacterium album* is 6.5–8.0, which could explain why OTU122 is not observed at the higher pH conditions. OTU2 was the most dominating OTU in Boom Clay borehole water at initial pH 9 and it was also present in the Harpur Hill sediment samples at pH 9 (Figure 8). Species from the *Acidovorax* genus have been identified and isolated from multiple piezometers in the HADES URF and also from Opalinus Clay borehole water at the Mont Terri URF (Wouters et al., 2013; Bleyen et al., 2017, 2021). In addition, the genus *Acidovorax* belongs to the family of *Comamonadaceae*, which are abundant in the Harpur Hill sediment (Smith et al., 2016). In general, *Acidovorax* is known to include metabolically versatile species and it was shown to have high nitrate reducing potential (Wouters et al., 2013). Our data indicate that it is rather sensitive to pH as it was almost completely outcompeted in the samples with an initial pH of 10.5. Although the relative abundance of OTU2 at pH 12.5 after 110 days is rather high and within the same range as the inoculum, the cell number was much lower and thus its absolute abundance was very low (Supplementary Figure 5). No nitrate reduction or acetate consumption was observed in those conditions so the species present were probably not active. Another member of the *Comamonadaceae* family, OTU3, classified as *Simplicispira*, is significantly correlated to the Harpur Hill sediment samples at an initial pH of 9 (Figure 8; Supplementary Table 1). The genus *Simplicispira* is known to be able to reduce nitrate and has been identified as one of the dominant genera in groundwater highly contaminated with nitrate (Grabovich et al., 2006; Safonov et al., 2018). At pH 10.5, OTU3 is outcompeted by another member of the *Comamonadaceae,* namely the genus *Hydrogenophaga* (represented by OTU6 and OTU15; Figure 8; Supplementary Figure 5). This genus has been identified to be present in the Harpur Hill sediment at high pH and also in other highly alkaline environments (Rizoulis et al., 2016; Smith et al., 2016). Members of the *Hydrogenophaga* comprise chemo-organothrophic and chemolithoautothrophic species. Several isolates were identified as nitrate reducers (Kämpfer et al., 2005; Mantri et al., 2016). OTU1, member of the *Pseudomonas* genus, is present in all samples of Boom Clay borehole water, which is not surprising as this genus is omnipresent in Boom Clay borehole water (Wouters et al., 2013; Honty and Mijnendonckx, 2022). It was also one of the dominant species present during and after nitrate injection tests in Opalinus Clay borehole water (Bleyen et al., 2017, 2021). In the present study, it is significantly correlated with the samples with an initial pH of 10.5 (Figure 8; Supplementary Table 1). Phosphate addition resulted in a substantial growth of *Pseudomonas* at pH 10.5, which was also observed by an increase in total cell count and a rapid consumption of acetate and nitrate (Supplementary Figures 4, 5). The high abundance of *Pseudomonas* after phosphate supplementation at pH 12.5 remains inconclusive as no data are present from immediately before phosphate addition. Nevertheless, although the pH decreased to pH 10 in the replicate of the Boom Clay borehole water community shown in Figure 8, no acetate or nitrate consumption was observed. Based on these results, it is unclear if the present microbial community is active. However, when a subsample of this replicate was diluted after 356 days in a liquid medium of pH 8.5 or 10.5, growth was observed after 1 week (Supplementary Figure 6), indicating that the community was still viable. Therefore, although the communities are unable to perform nitrate reduction, the long-term hyperalkaline conditions did not completely eliminate the microbial communities.

## Conclusion

We studied microbial nitrate reduction at three different pH levels to mimic the various locations in a deep geological nuclear waste repository for Eurobituminized waste in Belgium. It is expected that the cementitious materials used during geological disposal of this type of waste will induce high alkaline conditions resulting in a pH above 12.5 for thousands of years and afterwards gradually drops to pH 10. At the interface of Boom Clay with the disposal gallery, the pH will be not higher than pH 10.5. This alkaline plume reaches the first 1–3 m from the concrete, Boom Clay interface, while further in the Boom Clay, the pH will not change and will remain pH ~ 8.4 (Wang et al., 2007).

Our results indicate that nitrate reduction is not expected to occur at pH 12.5, not even in the presence of a pH adapted microbial community. However, the high pH did not completely eliminate the microbial community as cells could be resuscitated. Consequently, it seems that a high pH alone as stress factor will not eliminate the microbial presence in a geological repository, though it seems to provide enough stress to cause a significant shift in the microbial population and limit its nitrate reducing activity. However, several pH lowering processes (e.g., degradation of bitumen into CO_2_ or organic acids) can be present in a repository, which may result in small niches with lower pH (Valcke et al., 2022). Based on our data and on the available literature, microbial activity would be possible in those niches if pH < 11. Moreover, batch experiments with the denitrifying bacteria *Halomonas desiderata* focusing on the barrier between bituminized waste and the concrete overpack, showed that the presence of a solid cement fraction enhanced the denitrification rates and enabled denitrification at pH 10–12. This was accompanied by biofilm formation on the cement paste (Alquier et al., 2014). A follow-up study where a bioreactor was connected to an exposure chamber with cement paste examined various scenarios likely to occur in a geological repository, i.e., a nutrient limited environment. Here, it was shown that planktonic cells in the bioreactor were not able to carry out denitrification at pH 12, while denitrification was still ongoing in the biofilms formed at the cement surface in the exposure chamber (Rafrafi et al., 2017).

Based on the present study, it is clear that at the interface between the disposal gallery and the Boom Clay, the pH will not be sufficiently high to inhibit microbial nitrate reduction and acetate but also organic compounds leaching from bituminized waste can be used as electron donor to fuel nitrate reduction. These microbial reactions could have an impact on the redox conditions in the pore water. Increasing the pH causes differences in the predominance of nitrate reducing reactions and—in case acetate is present—seem to result in the accumulation of nitrite, either intermediately (for a pH adapted microbial community) or as a long-term end result (when the community is not adapted to a high pH). Nitrite is shown to be able to chemically oxidize redox active clay components which necessitates further studies explicating the impact of the microbial nitrate reduction processes on the mobility of redox-sensitive radionuclides. Complete denitrification to nitrogen gases with organic electron donors could lead to the formation of a separate gas phase, if the concentration of produced N gases would exceed the solubility limit of the gases. As clay formations are very gas-tight, gas evacuation is slow and an excessive gas pressure increase might cause fissuring of the host rock, which would result in the formation of preferential pathways for radionuclide migration (Mallants et al., 2007; Harrington et al., 2012).

Besides pH, the prevailing high salinity conditions closer to the bituminized waste monolith (up to few molars), will provide an additional stress to the microbial community. Maximum NO_3_^−^ concentrations are expected to reach between 0.5 and 1 M at the gallery interface, and decrease also fast within the host formation (e.g., 0.1 M at a distance of 5 m in the Boom Clay). These values are likely quite conservative due to the assumptions in the used model (Weetjens et al., 2010). Note that at high nitrate concentrations the presence of other nutrients and electron donors in the Boom Clay are expected to limit the denitrification reactions, and this on top of the stress induced by salinity and/or pH. Therefore, to further assess possible microbial presence in the near field of the disposal gallery, it would be interesting to study the effect of the high salinity and the combination of both stress factors on the microbial community.

## Data availability statement

The datasets generated and analyzed during the current study are available in the NCBI Sequence Read Archive (SRA) repository under project number PRJNA847218.

## Author contributions

KM and NB contributed to conception and design of the study. KM, IC, and AV performed the experimental work. KM performed the data analysis and wrote the first draft of the manuscript. NB performed a critical revision of the manuscript. All authors contributed to the article and approved the submitted version.

## Funding

The MIND-project has received funding from the European Union’s Euratom research and training program (Horizon2020) under grant agreement 661880.

## Conflict of interest

The authors declare that the research was conducted in the absence of any commercial or financial relationships that could be construed as a potential conflict of interest.

## Publisher’s note

All claims expressed in this article are solely those of the authors and do not necessarily represent those of their affiliated organizations, or those of the publisher, the editors and the reviewers. Any product that may be evaluated in this article, or claim that may be made by its manufacturer, is not guaranteed or endorsed by the publisher.
