# Supplementary material for: pH and microbial community determine the denitrifying activity in the presence of nitrate-containing radioactive waste
Source: Front Microbiol. 2022 Oct 21;13:968220. doi: 10.3389/fmicb.2022.968220 (PMC9634998; doi:10.3389/fmicb.2022.968220)
Supplement: Supplementary file 2 [file Data_Sheet_1.DOCX]

Supplementary Material

pH and microbial community determine the denitrifying activity in the presence of nitrate-containing radioactive waste

Kristel Mijnendonckx^1*^, Nele Bleyen^2^, Axel Van Gompel^3^, Ilse Coninx^1^, Natalie Leys^1^

^1^Unit of Microbiology, SCK CEN, Boeretang 200, Mol, Belgium

^2^W&D expert group, SCK CEN, Boeretang 200, Mol Belgium

^3^Biosphere Impact Studies, SCK CEN, Boeretang 200, Mol, Belgium

*** Correspondence:**Kristel Mijnendonckx
kmijnend@sckcen.be

Keywords: nitrate reduction, alkaline pH, Harpur Hill sediment, Boom Clay borehole water, bitumen (Min.5-Max. 8)

## Supplementary Figures


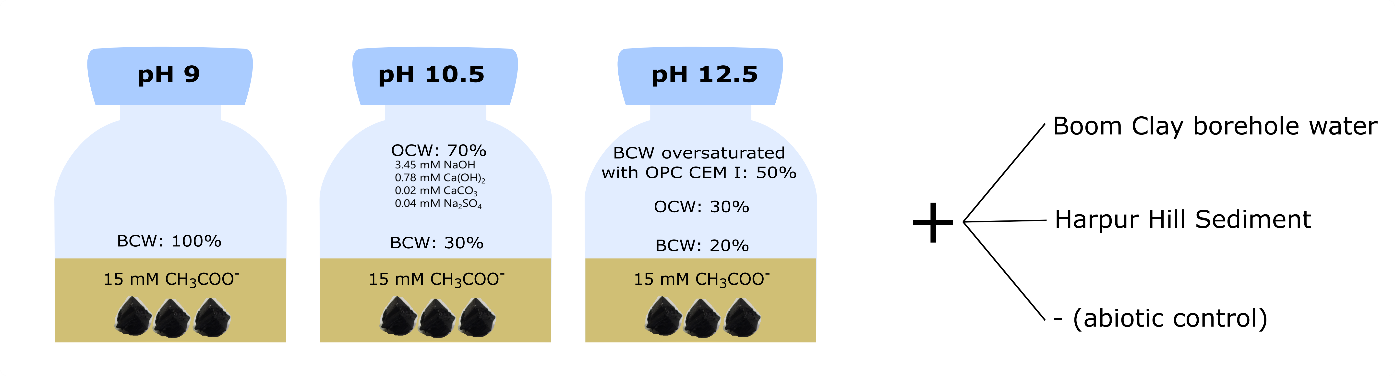


Supplementary Figure 1: Schematic representation of the different experimental conditions. BCW: filter sterilized and autoclaved Boom Clay water; OCW: Old Cement water; OPC CEM I: Ordinary Portlandite Cement CEM I. All conditions were prepared in triplicate.


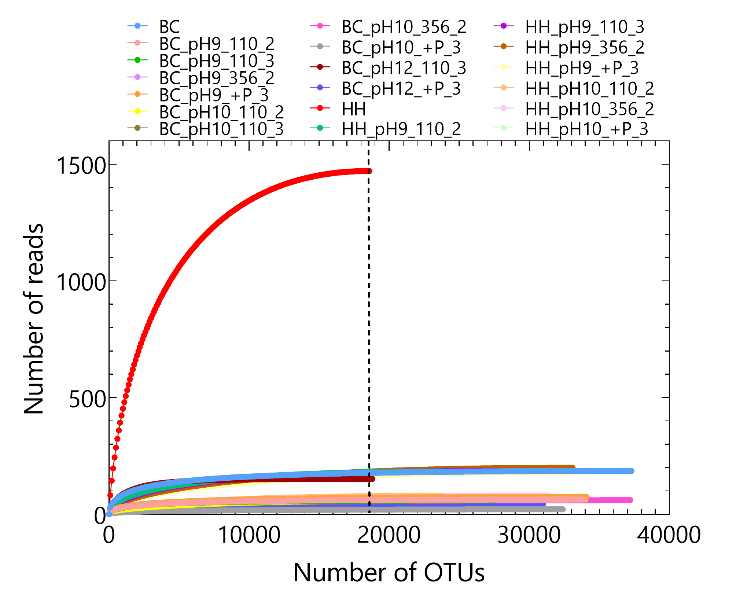


Supplementary Figure 2: Rarefaction curves showing the number of OTUs in function of the number of reads.


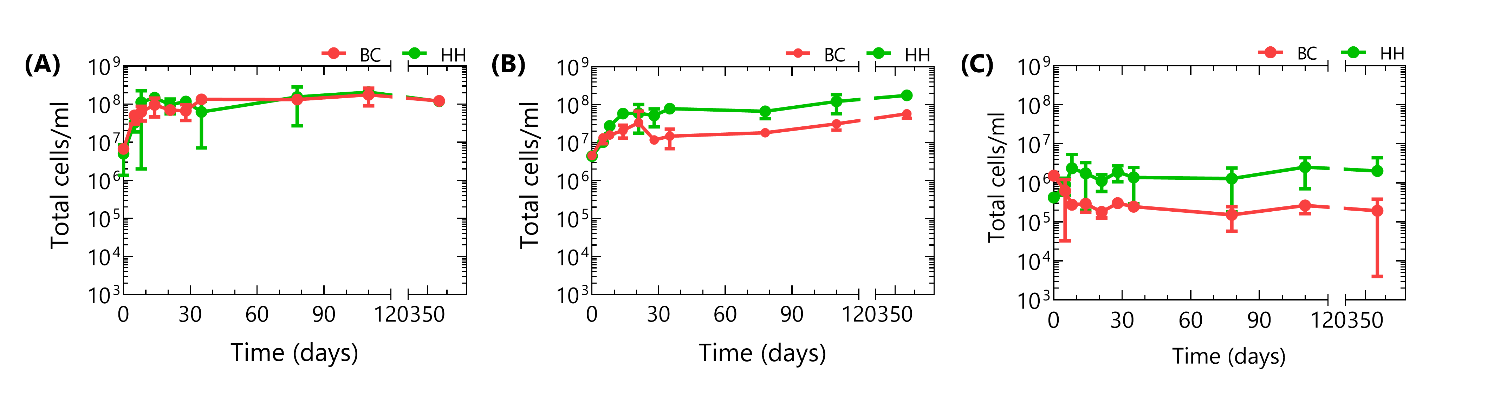


Supplementary Figure 3: Total cell concentration of the Boom Clay borehole water community (red) and the Harpur Hill sediment (green) determined with flow cytometry at **(A)** pH 9; **(B)** pH 10.5 and **(C)** pH 12.5. Values are the average and standard deviation of three replicates except for day 356 where only 2 replicates are included.


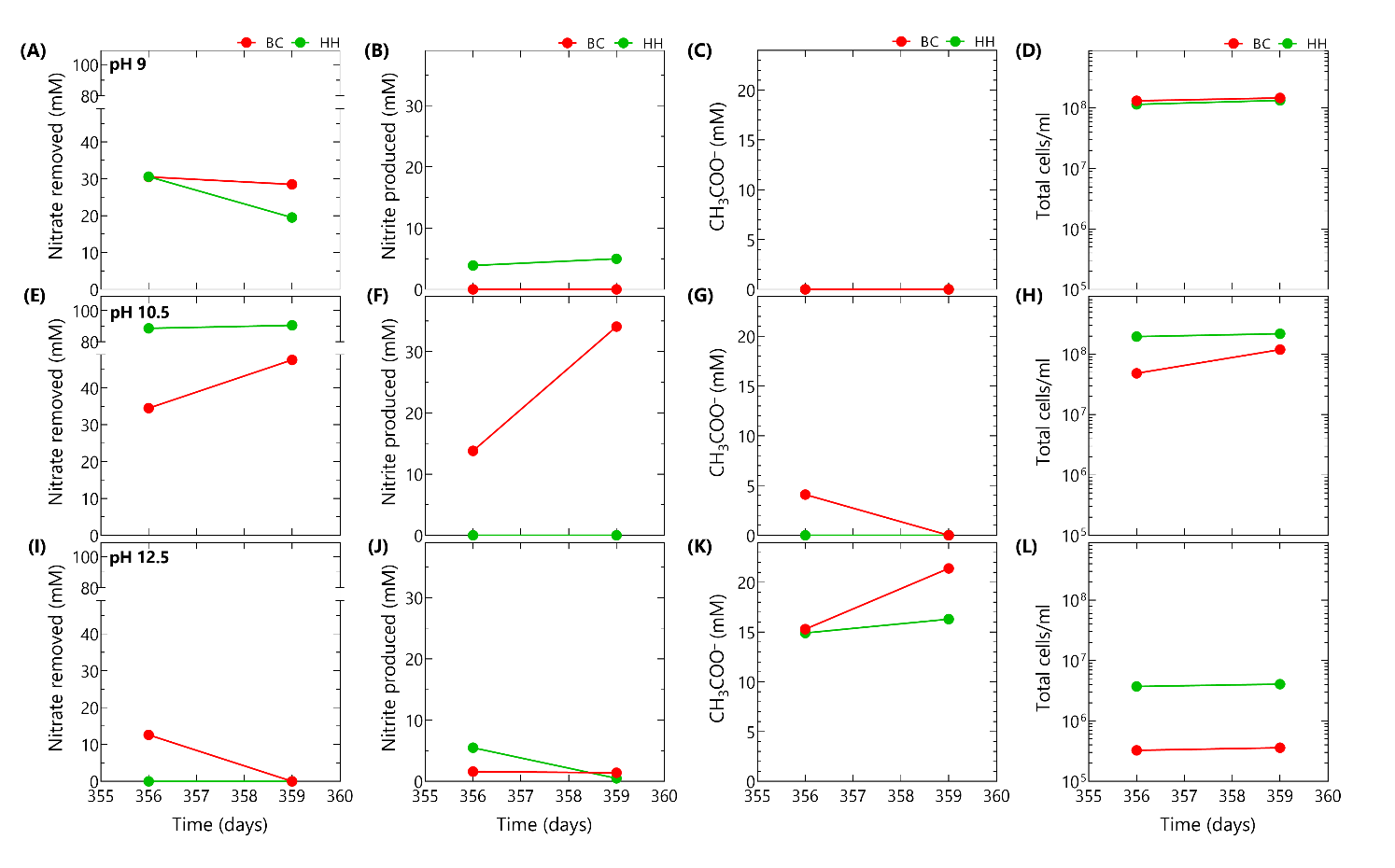


Supplementary Figure 4: The effect of the addition of phosphate on the nitrate removal **(A,E,I)**, nitrite production **(B,F,J)**, acetate consumption **(C,G,K)** and total cell concentration **(D, H, L)** at pH **(A-D)**, pH 10.5 **(E-H)** and pH 12.5 **(I-L)** for the Boom Clay borehole water (red) and the Harpur Hill sediment (green) microbial communities.


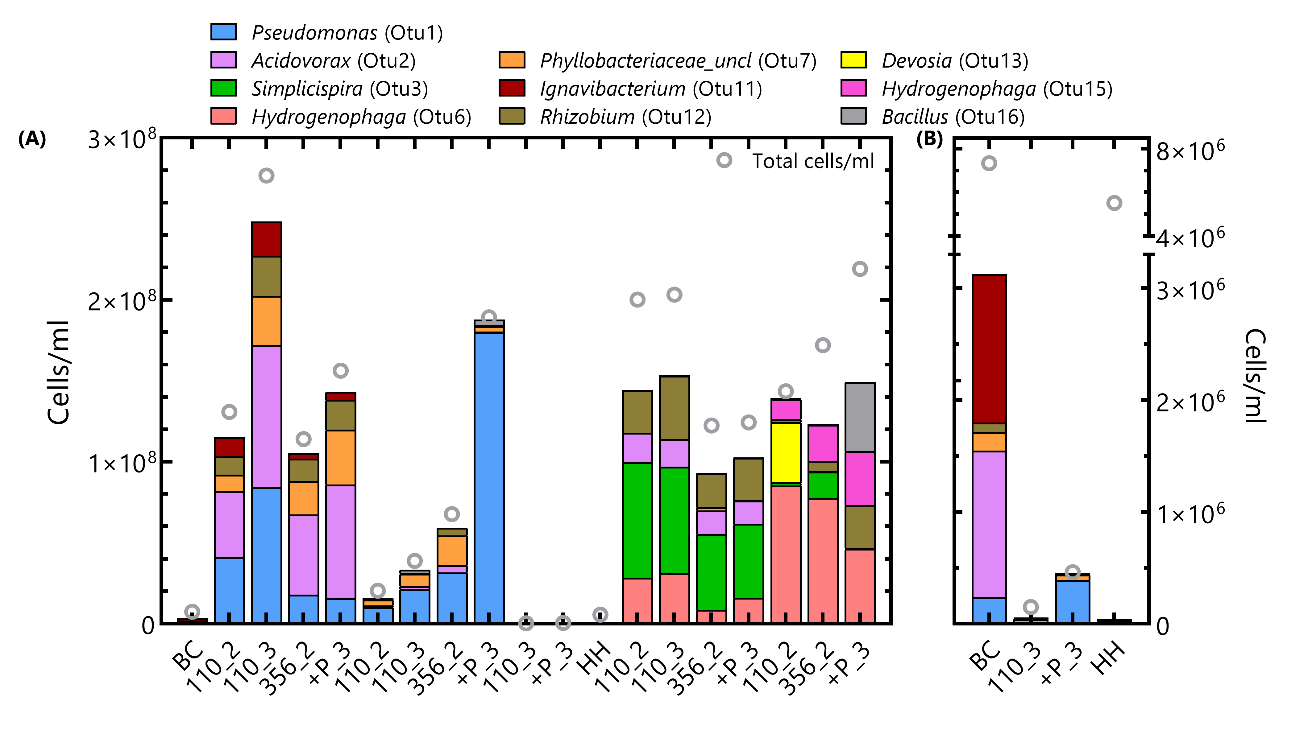


Supplementary Figure 5: Absolute OTU abundances in cells per ml based on flow cytometry measurements for **(A)** all samples and **(B)** samples with a lower cell number. The total cell number is presented by a grey circle.


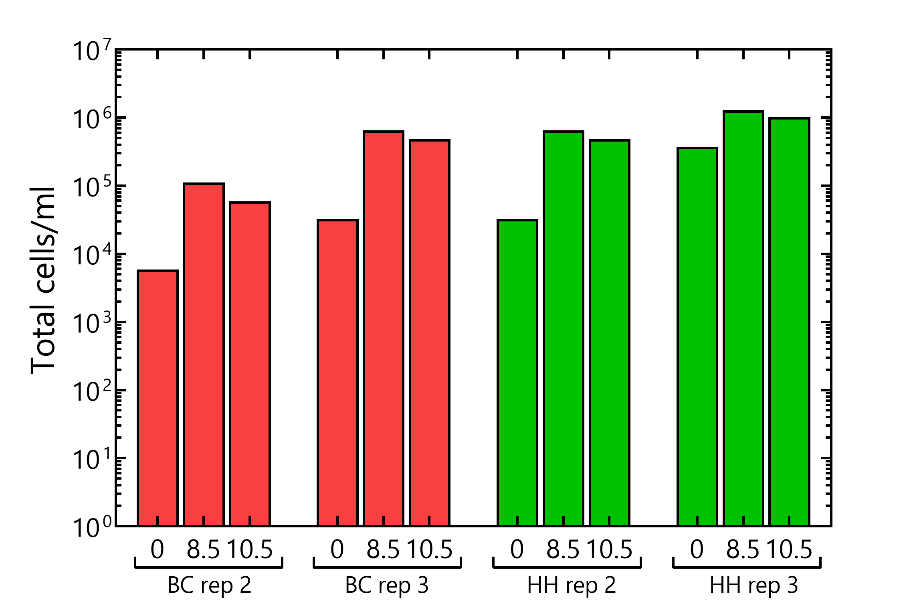


Supplementary Figure 6: Total cell concentration measured with flow cytometry after 1 week in medium of pH 8.5 (‘8.5’) and pH 10.5 (‘10.5’) after being exposed to pH 12.5 for 356 days (‘0’). Boom Clay borehole water samples are colored red and the Harpur Hill sediment is colored green. Two replicates of each community (‘rep 2’ and ‘rep 3’) were measured.
